# CD25^+^FOXP3^+^CD45RA^−^ regulatory T-cell infiltration as a prognostic biomarker for endometrial carcinoma

**DOI:** 10.1186/s12885-024-12851-0

**Published:** 2024-09-04

**Authors:** Asami Suto, Takeo Minaguchi, Nan Qi, Kaoru Fujieda, Hiroya Itagaki, Yuri Tenjimbayashi, Ayumi Shikama, Nobutaka Tasaka, Azusa Akiyama, Sari Nakao, Chigusa Nakahashi-Oda, Yusuke Kobayashi, Akira Shibuya, Toyomi Satoh

**Affiliations:** 1 Department of Obstetrics and Gynecology, Institute of Medicine, University of Tsukuba, 1-1-1 Tennoudai, Tsukuba, 305-8575 Ibaraki Japan; 2 Doctoral Program in Obstetrics and Gynecology, Graduate School of Comprehensive Human Sciences, University of Tsukuba, Tsukuba, Ibaraki Japan; 3 Department of Immunology, Institute of Medicine, University of Tsukuba, Tsukuba, Ibaraki Japan

**Keywords:** CD25, FOXP3, CD45RA, Treg cell, Survival, Endometrial carcinoma

## Abstract

**Background:**

Regulatory T (Treg) cells reportedly play crucial roles in tumor angiogenesis as well as antitumor immunity. In order to explore their therapeutic potential, we investigated the precise prognostic impact of Treg markers in endometrial carcinoma.

**Methods:**

We performed multiplexed immunofluorescence and quantitative image analyses of CD25, FOXP3, CTLA4, and CD45RA in tumor specimens from 176 consecutive patients treated at our institution for primary endometrial carcinomas. Bioinformatics analyses were further conducted to corroborate the findings.

**Results:**

High CD25^+^, FOXP3^+^, and CD25^+^FOXP3^+^CD45RA^−^ stromal cell counts correlated with better overall survival (OS) (*p* = 0.00019, 0.028 and 0.0012) and MSI-high (*p* = 0.015, 0.016 and 0.047). High CD45RA^+^ stromal cell count was associated with superficial myometrial invasion (*p* = 0.0038). Bioinformatics survival analysis by Kaplan-Meier plotter showed that high CD25, FOXP3, CTLA4, and CD45RA mRNA expressions correlated with better OS (*p* = 0.046, 0.00042, 0.000044, and 0.0022). Univariate and multivariate analyses with various clinicopathologic prognostic factors indicated that high CD25^+^ or CD25^+^FOXP3^+^CD45RA^−^ stromal cell count was significant and independent for favorable OS (*p* = 0.0053 and 0.0015). We subsequently analyzed the correlations between the multiplexed immunofluorescence results and treatment-free interval (TFI) after primary chemotherapy in recurrent cases, finding no significant associations. Further analysis revealed that high ratio of CD25^+^ : CD8^+^ cell count or CD25^+^FOXP3^+^CD45RA^−^ : CD8^+^ cell count correlated with longer TFI (*p* = 0.021 and 0.021).

**Conclusion:**

The current observations suggest that the balance between CD25^+^ or CD25^+^FOXP3^+^CD45RA^−^ cells and CD8^+^ cells, corresponding to promoting or inhibiting effect on tumor angiogenesis, affect tumor chemosensitivity leading to prognostic significance. CD25^+^FOXP3^+^CD45RA^−^ effector Treg tumor infiltration may serve as a useful prognostic biomarker and a potential target for immunotherapeutic manipulation of tumor chemosensitivity by novel management for advanced/recurrent endometrial carcinomas.

## Background

Endometrial cancer is the most common malignancy of the female reproductive organs in developed countries, and the incidence of the disease is recently increasing [1]. Majority of endometrial cancers are diagnosed in early stages, but 10–15% are in advanced-stage diseases [2], which are refractory due to resistance to chemotherapies and/or radiotherapies. In contrast with cervical cancer, to date no prophylactic measures have been established for endometrial cancer. In recent decades, a variety of molecular targeting agents have been clinically applied for the treatment of endometrial cancer including immune checkpoint inhibitors (ICIs) [3]. Combination of pembrolizumab, an anti-PD-1 antibody, and lenvatinib, a multi-kinase inhibitor, has been shown to be effective for advanced endometrial cancer irrespective of tumor mismatch repair (MMR) status [4]. Furthermore, a number of clinical trials investigating combinations of ICIs and various sorts of molecular targeting agents are currently ongoing for gynecological malignancies [5–9].

In the tumor microenvironment, cellular immunity is facilitated by CD8^+^ T cells which are activated by antigen-presenting cells recognizing tumor-derived neo-antigens. PD-L1 and PD-L2 expressed on the surface of tumor cells bind to PD-1 on activated CD8^+^ T cells and deactivate the anti-tumor immunity [10]. ICIs including pembrolizumab block the PD-1/PD-L1 interaction, and exert antitumor effect by reactivating the anti-tumor immunity. CD4^+^ T cells are derived from thymus, and are activated through T cell receptor (TCR) stimulation by such as infectious agents and tumors. Activated CD4^+^ T cells, or helper T cells, are classified as Th1, Th2, Th17, and regulatory T cell (Treg cell), etc. [11]. Th1, Th2, and Th17 activate cellular and humoral immunity, whereas Treg cells suppress them by inhibiting the other helper T cells. Upon TCR stimulation, naïve Treg cells (FOXP3^lo^CD45RA^+^CD25^lo^) are activated and differentiated into effector Treg cells (FOXP3^hi^CD45RA^−^CD25^hi^) acquiring strong immune suppressing ability [12]. Effector Treg cells inactivate CD8^+^ T cells through the mediation of various cytokines as well as the inhibition of antigen presenting cells by the interaction between CTLA4 and CD80/86 [12]. Anti-CTLA4 antibodies, another type of ICI, including ipilimumab reactivate CD8^+^ T cells and exert antitumor effect [13]. In addition to antitumor immunity, Treg cells are reported to be involved in regulating tumor angiogenesis in multiple malignancies as well [14–19].

We have previously focused on antitumor immunity in the microenvironment of endometrial cancer, and reported that high density of CD4^+^ tumor-infiltrating immune cells (TICs) was a significant and independent prognostic factor for favorable overall survival in endometrial cancers [20]. Based on this finding, we further explored the underlying pathways for the purpose of providing useful information for novel immunotherapeutic strategies for endometrial cancer.

## Methods

### Patients and specimens

All patients diagnosed with endometrial carcinoma, who received primary surgery at the University of Tsukuba Hospital between 1999 and 2009, were identified through our database. A total of consecutive 176 patients were included, and their medical records were retrospectively reviewed. Patients with carcinosarcomas or who received neoadjuvant chemotherapy were excluded. The study protocol was approved by the Ethics Committee of the University of Tsukuba Hospital. All specimens were obtained by the opt-out approach in accordance with the national legislation [21]. A median follow-up period excluding patients who died was 142 months (range, 3-259 months). Follow-up data were retrieved until 2023-8-4. Staging was conducted according to the criteria of International Federation of Gynecology and Obstetrics (FIGO, 2023) [22]. Molecular classifications were not integrated into staging due to assessment performed in not all patients. Treatment of patients was previously described [23]. The patient demographics are summarized in Table 1.

**Table 1:** Patient demographics

Characteristic			Number (*n* = 176)		%
Median age (range)			57 (26–84)		
FIGO stage^*^					
	I		98		56
		IA		74	42
		IB		22	13
		IC		2	1
	II		39		22
		IIA		11	6
		IIB		12	7
		IIC		16	9
	III		29		16
		IIIA		9	5
		IIIB		2	1
		IIIC		18	10
	IV		10		6
		IVA		0	0
		IVB		6	3
		IVC		4	2
Histotype					
	Endometrioid		161		91
		G1		94	53
		G2		49	28
		G3		18	10
	Serous		8		5
	Clear cell		2		1
	Undifferentiated		2		1
	Mixed epithelial		3		2
Myometrial invasion > 1/2			64		36
Lymphovascular space invasion			70		40
Primary treatment					
	Surgery		176		
		Lymphadenectomy		137	78
		Lymphnode sampling		16	9
		Lymphnode not removed		23	13
Adjuvant chemotherapy			43		
	TC			37	21
	CAP			4	2
	IAP			1	1
	C			1	1
Adjuvant radiotherapy			54		31
MSI	MSI-high		32		18
	MSI-low		5		3
	MSS		139		79

Abbreviations: *FIGO* International Federation of Gynecology and Obstetrics, *TC* paclitaxel and carboplatin combination, *CAP* cyclophosphamide, doxorubicin, and cisplatin combination, *IAP* ifosfamide, doxorubicin, and cisplatin combination, *C* carboplatin, *MSI* microsatellite instability, *MSS* microsatellite stable

^*^Molecular classifications were not integrated due to assessment performed in not all patients

### Multiplexed immunofluorescence

Multiplexed immunofluorescence staining was performed using Opal Multiplex IHC kit (Akoya Biosciences, Marlborough, MA, USA). We selected the slides including the most abundant tumor tissues without necrosis, typically near the center of the tumors, by reviewing the corresponding hematoxylin and eosin staining slides. Tissue slides were deparaffinized and rehydrated, and antigen was retrieved by microwave treatment in AR9 buffer (PerkinElmer, Waltham, MA, USA). The following sequential steps were repeated for each protein: blocking with antibody diluent (PerkinElmer), incubation with primary antibody, detection with Opal Polymer HRP Ms + Rb secondary antibody (PerkinElmer) or rabbit anti-goat IgG (H + L) secondary antibody (Vector Laboratories, Newark, CA, USA), signal generation with Opal tyramide signal amplification (TSA) plus agent, and heating by microwave treatment in AR6 or AR9 buffer (PerkinElmer). The primary antibodies and corresponding TSA used for each protein were as follows: CD25 (EPR6452, rabbit monoclonal, Abcam, Cambridge, UK) and Opal 650, FOXP3 (236 A/E7, mouse monoclonal, Invitrogen, Carlsbad, CA, USA) and Opal 570, CTLA4 (Q6GR94, goat polyclonal, R&D Systems, Minneapolis, MN, USA) and Opal 520, and CD45RA (HI100, mouse monoclonal, Invitrogen) and Opal 480. Nuclei were visualized with DAPI.

### Multispectral imaging and quantitative image analysis

**Fig. 1:**
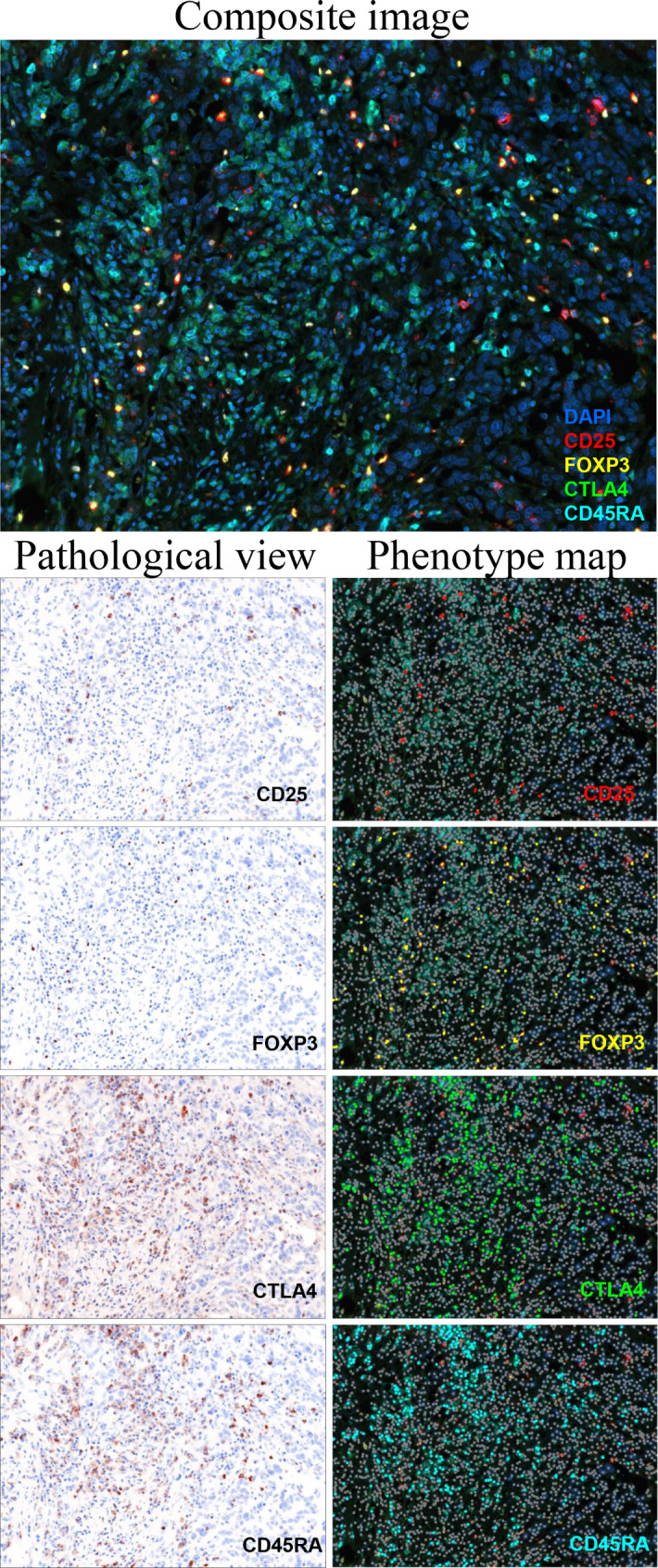
Representative images of multiplexed immunofluorescence analysis. Composite image, pathological views, and phenotype maps for CD25, FOXP3, CTLA4, and CD45RA. × 200

Each fluorescence was detected by a Mantra fluorescence microscope (Akoya Biosciences) and evaluated using the inForm image analysis software (version 2.6.0, Akoya Biosciences). Upon analyzing the images acquired with a Mantra system, fluorescence intensities of each fluorophore were normalized to exposure time in the inForm software. Spectral library slides each stained with a single fluorophore using Cytokeratin 7 (MAA556Hu22, mouse monoclonal, Cloud-Clone Corp., Katy, TX, USA) were used as the reference for fluorescent target quantification. Quantitative image analyses were done by unmixing multispectral images using the spectral libraries, followed by tissue segmentation of manually selecting tumor and stromal categories in the tissue, cell segmentation of identifying single cells, and active learning phenotyping. The phenotyping data produced by inForm were processed by phenoptrReports tool (Akoya Biosciences) upon RStudio software (version 2022.07.2–576, https://dailies.rstudio.com/). Five fields at magnification of ×200 were randomly selected from each slide, but excluding sites showing dominantly tumor or stromal compartments, and average counts of positive cells were calculated. For the purpose of examining tumor-infiltrating lymphocytes, we evaluated specifically the stromal compartments. Representative images of multiplexed immunofluorescence analysis are depicted in Fig. 1.

### Immunohistochemistry

Immunohistochemical staining and counting of CD8^+^ TICs were conducted as described previously [20].

### Microsatellite instability (MSI) analysis

MSI status of tumors was analyzed as previously reported [24].

### Bioinformatics analysis

Survival analyses according to the mRNA expressions were performed by Kaplan-Meier plotter (https://kmplot.com/analysis/) [25]. Survival analyses based on tumor immune infiltrate subsets and gene expressions were done by TIMER2.0 (http://timer.comp-genomics.org/timer/) [26, 27] which computationally infers the abundance of TIC subsets in tumors utilizing the data from The Cancer Genome Atlas (TCGA) [28].

### Statistical analyses

Differences in continuous variables were evaluated by the Mann-Whitney U test. Differences in proportions were evaluated by the Fisher’s exact test. The optimal cut-off values of cell counts according to multiplexed immunofluorescence results for the relationships with patient survival were calculated (Table 2), and Kaplan-Meier survival curves were created and compared by the log-rank test. The univariate and multivariate analyses were performed by the Cox proportional hazard model. Overall survival (OS) was defined as the interval between the start of primary treatment and death from any cause. Treatment-free interval (TFI) was defined as the interval between the end of primary adjuvant chemotherapy and the diagnosis of recurrence. R version 4.2.2 (https://www.r-project.org/) was used for all statistical analyses. *P*-values < 0.05 were considered as statistically significant.

**Table 2:** Optimal cut-off values of cell counts according to multiplexed immunofluorescence evaluations for the relationship with OS

	Mean ± SD	Min	Max	Cut-off	Category	N (%)
CD25^+^ stromal cells	17.3 ± 20.2	0.2	123.2	1.8	High cell counts	154 (88)
					Low cell counts	22 (13)
FOXP3^+^ stromal cells	32.1 ± 31.3	0.6	190.6	64.4	High cell counts	23 (13)
					Low cell counts	153 (87)
CTLA4^+^ stromal cells	30.4 ± 103.4	0.0	1227.2	18.6	High cell counts	49 (28)
					Low cell counts	127 (72)
CD45RA^+^ stromal cells	112.0 ± 145.7	0.0	993.8	231.8	High cell counts	26 (15)
					Low cell counts	150 (85)
CD25^+^FOXP3^+^CD45RA^−^ stromal cells	7.7 ± 8.1	0.0	53.4	1.6	High cell counts	143 (81)
					Low cell counts	33 (19)
CD8^+^TIC^*^	203.9 ± 122.7	22.0	582.7	298.7	High cell counts	36 (20)
					Low cell counts	140 (80)

Abbreviation: *OS* overall survival, *SD* standard deviation, *Min* minimum, *Max* maximum, *TIC* tumor-infiltrating immune cell

^*^CD8^+^ TICs were counted by immunohistochemistry

## Results

**Fig. 2:**
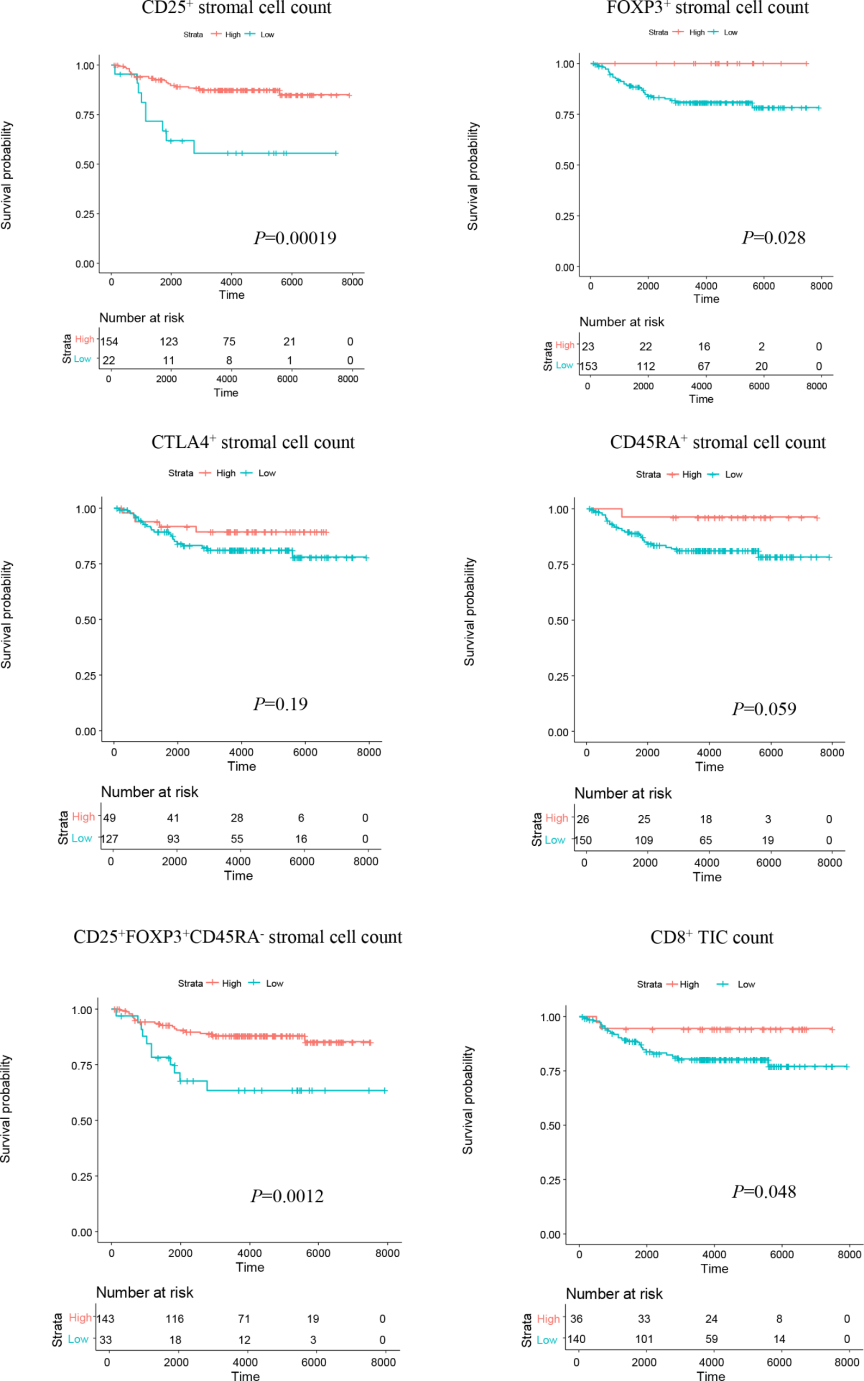
Comparison of overall survival curves (days) of patients with endometrial carcinomas according to CD25^+^, FOXP3^+^, CTLA4^+^, CD45RA^+^, or CD25^+^FOXP3^+^CD45RA^−^ stromal cell counts, or CD8^+^ tumor-infiltrating immune cell (TIC) counts

We performed multiplexed immunofluorescence and quantitative image analysis for Treg markers, CD25, FOXP3, CTLA4, and CD45RA, in tumor tissues from 176 patients with endometrial carcinomas (Table 2). We first examined mutual relationships among the multiplexed immunofluorescence results. High CD25^+^ stromal cell count was significantly associated with high CTLA4^+^ stromal cell count, and high FOXP3^+^ stromal cell count was significantly associated with high CD45RA^+^ stromal cell count (*p* = 0.00062 and 0.00029; Table 3). No other significant correlations were observed among the marker-positive stromal cell counts. We next analyzed survival curves according to the multiplexed immunofluorescence results. CD25^+^, FOXP3^+^, and CD25^+^FOXP3^+^CD45RA^−^ stromal cell counts were all significantly associated with better OS (*p* = 0.00019, 0.028, and 0.0012; Fig. 2). Next, univariate analyses of prognostic factors for OS were conducted. CD25^+^ and CD25^+^FOXP3^+^CD45RA^−^ stromal cell counts were both significantly associated with better OS, while old age, advanced stage, deep myometrial invasion, and present lymphovascular space invasion were all significantly associated with worse OS (*p* = 0.00058, 0.0023, 0.000012, 4.3E-08, 0.00033, and 0.0015; Table 4). Subsequent multivariate analysis using CD25^+^ stromal cell count and clinicopathological prognostic factors indicated that CD25^+^ stromal cell count was a significant and independent favorable prognostic factor, and that old age and advanced stage were significant and independent unfavorable prognostic factors (*p* = 0.0053, 0.00094, and 0.0014; Table 4). Likewise, multivariate analysis using CD25^+^FOXP3^+^CD45RA^−^ stromal cell count and clinicopathological prognostic factors indicated that CD25^+^FOXP3^+^CD45RA^−^ stromal cell count was a significant and independent favorable prognostic factor, and that old age and advanced stage were significant and independent unfavorable prognostic factors (*p* = 0.0015, 0.00097, and 0.0010; Table 4).

**Table 3:** Mutual relationships among stromal cell counts based on multiplexed immunofluorescence

	FOXP3^+^ stromal cell count			CTLA4^+^ stromal cell count			CD45RA^+^ stromal cell count		
	High (n = 23)	Low (n = 153)	*P*-value	High (n = 49)	Low (n = 127)	*P*-value	High (n = 26)	Low (n = 150)	*P*-value
High CD25^+^ stromal cell count	23 (100%)	131 (86%)	0.083	49 (100%)	105 (83%)	0.00062	24 (92%)	130 (87%)	0.54
High FOXP3^+^ stromal cell count	-	-	-	10 (20%)	13 (10%)	0.084	10 (38%)	13 (9%)	0.00029
High CTLA4^+^ stromal cell count	-	-	-	-	-	-	11 (42%)	38 (25%)	0.096

Abbreviations: *TIC* tumor-infiltrating immune cell

**Table 4:** Univariate and multivariate analyses of prognostic factors for OS

	Univariate				Multivariate						
	HR	95% CI	*P*-value		HR	95% CI	*P*-value		HR	95% CI	*P*-value
High CD25^+^ stromal cell count	0.25	0.11–0.55	0.00058		0.31	0.13–0.70	0.0053		-	-	-
High FOXP3^+^ stromal cell count	1.2E-08	0-Inf	1.0		-	-	-		-	-	-
High CTLA4^+^ stromal cell count	0.53	0.20–1.4	0.20		-	-	-		-	-	-
High CD45RA^+^ stromal cell count	0.18	0.025-1.3	0.093		-	-	-		-	-	-
High CD25^+^FOXP3^+^CD45RA^−^ stromal cell count	0.31	0.14–0.66	0.0023		-	-	-		0.28	0.12–0.61	0.0015
Old age (> 74 vs. ≤ 74)	6.0	2.7–13.4	0.000012		4.3	1.8–10.2	0.00094		4.2	1.8–9.9	0.00097
Advanced stage (III-IV vs. I-II)	8.8	4.0-19.1	4.3E-08		4.2	1.8–10.3	0.0014		4.4	1.8–10.7	0.0010
Deep myometrial invasion (> 1/2 vs. ≤ 1/2)	4.3	1.9–9.5	0.00033		1.6	0.66-4.0	0.29		1.8	0.73–4.5	0.20
LVSI (present vs. absent)	3.6	1.6-8.0	0.0015		2.2	0.91–5.2	0.080		2.3	0.97–5.7	0.058

Abbreviations: *OS* overall survival, *HR* hazard ratio, *CI* confidence interval, *Inf* infinity, *LVSI* lymphovascular space invasion

**Fig. 3:**
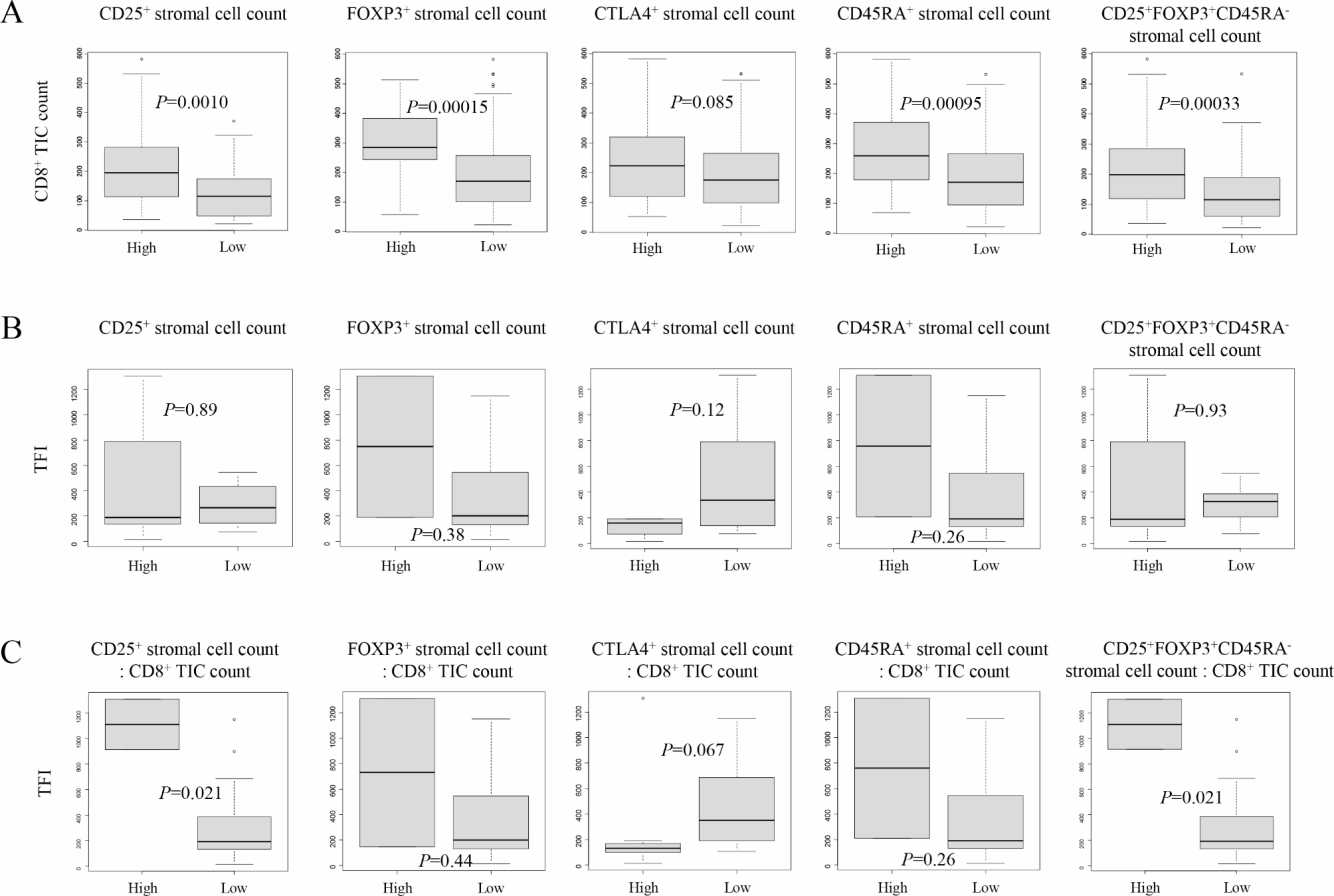
**A**, Comparison of CD8^+^ tumor-infiltrating immune cell (TIC) count of endometrial carcinoma tissues according to multiplexed immunofluorescence results. High (*n* = 154) vs. low (*n* = 22) CD25^+^ stromal cell count, high (*n* = 23) vs. low (*n* = 153) FOXP3^+^ stromal cell count, high (*n* = 49) vs. low (*n* = 127) CTLA4^+^ stromal cell count, high (*n* = 26) vs. low (*n* = 150) CD45RA^+^ stromal cell count, and high (*n* = 143) vs. low (*n* = 33) CD25^+^FOXP3^+^CD45RA^−^ stromal cell count. **B**, Comparison of treatment-free interval (TFI, days) of patients with endometrial carcinomas according to multiplexed immunofluorescence results. High (*n* = 16) vs. low (*n* = 4) CD25^+^ stromal cell count, high (*n* = 2) vs. low (*n* = 18) FOXP3^+^ stromal cell count, high (*n* = 4) vs. low (*n* = 16) CTLA4^+^ stromal cell count, high (*n* = 2) vs. low (*n* = 18) CD45RA^+^ stromal cell count, and high (*n* = 15) vs. low (*n* = 5) CD25^+^FOXP3^+^CD45RA^−^ stromal cell count. **C**, Comparison of TFI (days) of patients with endometrial carcinomas according to multiplexed immunofluorescence results. High (*n* = 2) vs. low (*n* = 18) ratio of CD25^+^ stromal cell count : CD8^+^ TIC count, high (*n* = 2) vs. low (*n* = 18) FOXP3^+^ stromal cell count : CD8^+^ TIC count, high (*n* = 7) vs. low (*n* = 13) CTLA4^+^ stromal cell count : CD8^+^ TIC count, high (*n* = 2) vs. low (*n* = 18) CD45RA^+^ stromal cell count : CD8^+^ TIC count, and high (*n* = 2) vs. low (*n* = 18) CD25^+^FOXP3^+^CD45RA^−^ stromal cell count : CD8^+^ TIC count

We next examined the relationships between stromal cell counts based on the multiplexed immunofluorescence and various clinicopathological factors. High CD45RA^+^ stromal cell count was significantly associated with superficial myometrial invasion (*p* = 0.0038; Table 5). High CD25^+^, FOXP3^+^, and CD25^+^FOXP3^+^CD45RA^−^ stromal cell counts were all significantly associated with MSI-high (*p* = 0.015, 0.016 and 0.047; Table 5). We further examined those relationships on the ratios of each stromal cell counts to total CD8^+^ cell counts. High ratios of CD25^+^ stromal cell count to total CD8^+^ cell count was significantly associated with young age and superficial myometrial invasion (*p* = 0.046 and 0.032; Table 6). High ratios of CD45RA^+^ stromal cell count to total CD8^+^ cell count was significantly associated with early stage and superficial myometrial invasion (*p* = 0.025 and 0.0016; Table 6). High ratios of CD25^+^FOXP3^+^CD45RA^−^ stromal cell count to total CD8^+^ cell count was significantly associated with young age and early stage (*p* = 0.048 and 0.046; Table 6). We further examined the correlations between stromal cell counts based on the multiplexed immunofluorescence and total CD8^+^ cell counts. High CD25^+^, FOXP3^+^, CD45RA^+^, and CD25^+^FOXP3^+^CD45RA^−^ stromal cell counts were all significantly associated with high CD8^+^ cell counts (*p* = 0.0010, 0.00015, 0.00095, and 0.00033; Fig. 3A).

**Table 5:** Relationships between stromal cell counts based on multiplexed immunofluorescence and clinicopathological factors

	CD25^+^ stromal cell count			FOXP3^+^ stromal cell count			CTLA4^+^ stromal cell count			CD45RA^+^ stromal cell count			CD25^+^FOXP3^+^CD45RA^−^ stromal cell count		
	High (n = 154)	Low (n = 22)	*P*-value	High (n = 23)	Low (n = 153)	*P*-value	High (n = 49)	Low (n = 127)	*P*-value	High (n = 26)	Low (n = 150)	*P*-value	High (n = 143)	Low (n = 33)	*P*-value
Old age (> 74 vs. ≤ 74)	15 (10%)	4 (18%)	0.27	1 (4%)	18 (12%)	0.47	4 (8%)	15 (12%)	0.60	1 (4%)	18 (12%)	0.32	13 (9%)	6 (18%)	0.21
Non-endometrioid (vs. Endometrioid)	11 (7%)	4 (18%)	0.1	3 (13%)	12 (8%)	0.42	5 (10%)	10 (8%)	0.76	3 (12%)	12 (8%)	0.46	10 (7%)	5 (15%)	0.16
Advanced stage (III/IV vs. I/II)	31 (20%)	8 (36%)	0.10	2 (9%)	37 (24%)	0.11	9 (18%)	30 (24%)	0.55	4 (15%)	35 (23%)	0.45	29 (20%)	10 (30%)	0.25
Deep myometrial invasion (> 1/2 vs. ≤ 1/2)	54 (35%)	10 (46%)	0.35	7 (30%)	57 (37%)	0.64	14 (29%)	50 (39%)	0.22	3 (12%)	61 (41%)	0.0038	53 (37%)	11 (33%)	0.84
LVSI (present vs. absent)	61 (40%)	9 (6%)	1	10 (44%)	60 (39%)	0.82	23 (47%)	47 (37%)	0.23	10 (39%)	60 (40%)	1	60 (42%)	10 (30%)	0.24
MSI (MSI-high vs. MSI-low + MSS)	32 (21%)	0 (0%)	0.015	9 (39%)	23 (15%)	0.016	10 (20%)	22 (17%)	0.67	6 (23%)	26 (17%)	0.58	30 (21%)	2 (6%)	0.047

Abbreviations: *LVSI* lymphovascular space invasion, *MSI* microsatellite instability, *MSS* microsatellite stable

**Table 6:** Relationships between the ratios of stromal cell counts based on multiplexed immunofluorescence to CD8^+^ TIC count and clinicopathological factors

	CD25^+^ stromal cell count : CD8^+^ TIC count^*^			FOXP3^+^ stromal cell count : CD8^+^ TIC count^*^			CTLA4^+^ stromal cell count : CD8^+^ TIC count^*^			CD45RA^+^ stromal cell count : CD8^+^ TIC count^*^			CD25^+^FOXP3^+^CD45RA^−^ stromal cell count : CD8^+^ TIC count^*^		
	High (n = 28)	Low (n = 148)	*P*-value	High (n = 24)	Low (n = 152)	*P*-value	High (n = 68)	Low (n = 108)	*P*-value	High (n = 36)	Low (n = 140)	*P*-value	High (n = 27)	Low (n = 149)	*P*-value
Old age (> 74 vs. ≤ 74)	0 (0%)	19 (13%)	0.046	0 (0%)	19 (13%)	0.080	8 (12%)	11 (10%)	0.81	1 (3%)	18 (13%)	0.13	0 (0%)	19 (13%)	0.048
Non-endometrioid (vs. Endometrioid)	2 (7%)	13 (9%)	1	3 (13%)	12 (8%)	0.44	7 (10%)	8 (7%)	0.58	2 (6%)	13 (9%)	0.74	2 (7%)	13 (9%)	1
Advanced stage (III/IV vs. I/II)	3 (11%)	36 (24%)	0.14	3 (13%)	36 (24%)	0.29	13 (19%)	26 (24%)	0.46	3 (8%)	36 (26%)	0.025	2 (7%)	37 (25%)	0.046
Deep myometrial invasion (> 1/2 vs. ≤ 1/2)	5 (18%)	59 (40%)	0.032	6 (25%)	58 (38%)	0.26	22 (32%)	42 (39%)	0.42	5 (14%)	59 (42%)	0.0016	7 (26%)	57 (38%)	0.28
LVSI (present vs. absent)	9 (32%)	61 (41%)	0.41	8 (33%)	62 (41%)	0.65	28 (41%)	42 (39%)	0.87	11 (31%)	59 (42%)	0.25	12 (44%)	58 (39%)	0.67
MSI (MSI-high vs. MSI-low + MSS)	4 (14%)	28 (19%)	0.79	3 (13%)	29 (19%)	0.58	11 (16%)	21 (19%)	0.69	8 (22%)	24 (17%)	0.47	5 (19%)	27 (18%)	1

Abbreviations: *TIC* tumor-infiltrating immune cell, *LVSI* lymphovascular space invasion, *MSI* microsatellite instability, *MSS* microsatellite stable

^*^CD8^+^ TICs were counted by immunohistochemistry

We subsequently analyzed correlations between TFI and multiplexed immunofluorescence results. None of the stromal cell counts was found to be significantly associated with TFI (Fig. 3B). We further extended the analyses to the ratios of stromal cell counts to total CD8^+^ cell counts. The high ratios on CD25^+^ and CD25^+^FOXP3^+^CD45RA^−^ were both found to be significantly associated with long TFI (*p* = 0.021 and 0.021; Fig. 3C).

**Fig. 4:**
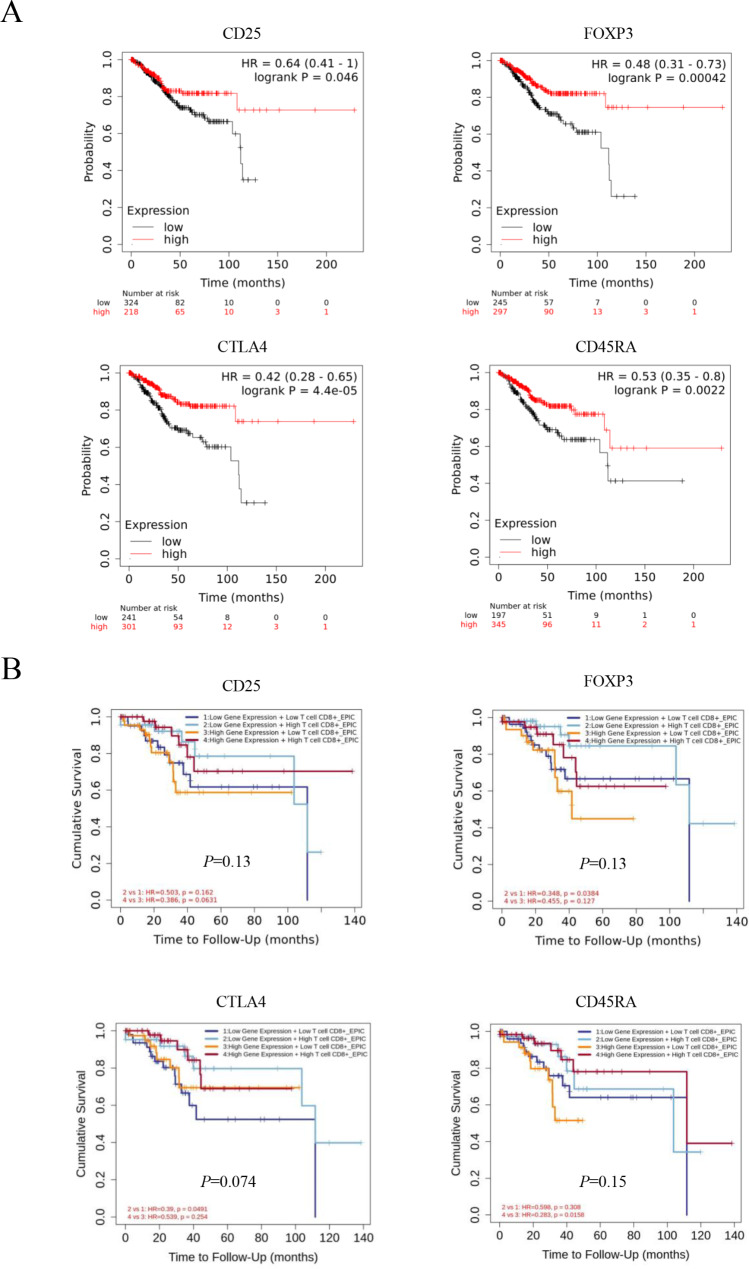
**A**, Kaplan-Meier plotter analysis of overall survival curves in patients with endometrial carcinomas according to the mRNA expressions. Survival curves of patients (*n* = 542) with high vs. low CD25 expression, high vs. low FOXP3 expression, high vs. low CTLA4 expression, and high vs. low CD45RA expression. **B**, TIMER2.0 analysis of overall survival curves in patients with endometrial carcinomas (*n* = 545) based on CD8^+^ T-cell infiltration and the gene expression level of CD25, FOXP3, CTLA4, or CD45RA

Lastly, we progressed to bioinformatics analyses by web tools. Kaplan-Meier survival curves according to mRNA expressions indicated that high expressions of CD25, FOXP3, CTLA4, and CD45RA all significantly correlated with better OS (*p* = 0.046, 0.00042, 0.000044, and 0.0022; Fig. 4A). We further conducted survival analyses by TIMER2.0 [26] based on CD8^+^ T-cell infiltration and gene expression levels. Tumors with low CD25 expression and high CD8^+^ T-cell infiltration showed the best OS, followed by those with high CD25 and high CD8^+^ T cell and those with low CD25 and low CD8^+^ T cell, whereas tumors with high CD25 and low CD8^+^ T cell showed the worst OS (Fig. 4B). FOXP3 showed the same survival profile as CD25 (Fig. 4B). CTLA4 showed a similar survival profile, being different in that tumors with low CTLA4 and low CD8^+^ T cell showed the worst OS, followed by those with high CTLA4 and low CD8^+^ T cell (Fig. 4B). Likewise, CD45RA showed a similar survival profile, but being different in that tumors with high CD45RA and high CD8^+^ T cell showed the best OS, followed by those with low CD45RA and high CD8^+^ T cell (Fig. 4B).

## Discussion

Our survival analyses based on the multiplexed immunofluorescence evaluations suggest that CD25^+^, FOXP3^+^, and CD25^+^FOXP3^+^CD45RA^−^ stromal cells have favorable prognostic effects (Fig. 2). Furthermore, the univariate and multivariate analyses indicate that CD25^+^ and CD25^+^FOXP3^+^CD45RA^−^ stromal cell counts were both significant and independent prognostic factors for favorable OS when each analyzed separately (Table 4). The subsequent analyses on the relationships with clinicopathological factors indicated that only CD45RA^+^ stromal cell count was significantly associated with a prognostic factor significant in the univariate analysis (Table 5), i.e. superficial myometrial invasion (Table 4), providing no speculation for prognostic functions of CD25^+^ and CD25^+^FOXP3^+^CD45RA^−^ cells. In order to explore possible explanations for prognostic functions of CD25^+^ and CD25^+^FOXP3^+^CD45RA^−^ cells, we next examined the correlations between TFI and the multiplex immunofluorescence evaluations. However, none of the stromal cell counts was found to be significantly associated with TFI (Fig. 3B). Further analyzing the correlations between TFI and the ratios of stromal cell counts to total CD8^+^ cell counts showed that TFI was significantly longer in tumors with high ratios of CD25^+^ or CD25^+^FOXP3^+^CD45RA^−^ stromal cell counts compared with tumors with low ratios (Fig. 3C). This observation suggests that these ratios may be related to tumor chemosensitivity, eventually affecting patient prognosis, as TFI is known to be associated with response to chemotherapy for recurrence and/or survival after recurrence in endometrial cancer [29–31].

CD25 and FOXP3 are known to have inactivating functions against CD8^+^ T cells [32]. Indeed, the survival analyses by TIMER2.0 [26–28], which computationally predicts the abundance of TIC subsets in tumors from the TCGA data, suggested that high CD8^+^ T-cell infiltration has tumor-suppressing effect, and that high CD25 and FOXP3 expressions both have antagonizing effects against that, resulting in the opposite prognostic significances (Fig. 4B). Against our expectation however, the survival analyses by multiplexed immunofluorescence showed that high CD25^+^, FOXP3^+^, and CD45RA^+^ stromal cell counts all correlated with better OS (Fig. 2). Keeping in line with and supporting these findings, the bioinformatics analyses by Kaplan-Meier plotter [25] also indicated that high expressions of CD25, FOXP3, and CD45RA significantly correlated with better OS (Fig. 4A). These results by Kaplan-Meier plotter as well as ours were reverse to what was expected considering the functions of immune cellular components. It is presumed that the results of our multiplexed immunofluorescence analyses may rather correspond to the stromal responses reflecting antitumoral immune status. This hypothesis may be supported by our finding that high CD25^+^, FOXP3^+^, and CD25^+^FOXP3^+^CD45RA^−^ stromal cell counts were all significantly associated with both high CD8^+^ TIC count (Fig. 3A) and MSI-high (Table 5), as MSI is known to cause high tumor mutation burden (TMB), leading to activation of antitumor immunity [33]. Moreover, the ratios of CD25^+^ or CD25^+^FOXP3^+^CD45RA^−^ stromal cell counts to CD8^+^ cell count were both found to significantly correlate with longer TFI (Fig. 3C). We speculate that CD25^+^ or CD25^+^FOXP3^+^CD45RA^−^ stromal cell count reflects the stromal responses to antitumoral immunity, and that their ratios to CD8^+^ cell count may be equivalent to the strength of antitumoral immune status particularly involved in tumor chemosensitivity. Additionally, another possible explanation is that the ratio of CD25^+^ or CD25^+^FOXP3^+^CD45RA^−^ stromal cell count to CD8^+^ cell count corresponds to the balance of promoting/inhibiting tumor angiogenesis. CD8^+^ T cells are known to inhibit tumor angiogenesis by secreting IFN-γ [19, 34], whereas the presence of CD25^+^/FOXP3^+^ tumor-infiltrating lymphocytes reportedly correlates with promoting intratumoral angiogenesis in multiple sorts of cancer including endometrial cancer [14–16]. These reports may explain the underlying mechanism for our finding, as tumor angiogenic status is considered to be involved in chemosensitivity. Besides, FOXP3^+^ Treg cell infiltration is generally associated with favorable prognosis in colorectal cancers [35], and high infiltration of Th17 cells was reportedly linked with poor prognosis in this disease [36]. Treg cells are suggested to inhibit protumorigenic effects by suppressing proinflammatory and tumor-promoting abilities of Th17 cells [37], providing a possible explanation for the favorable prognostic role of FOXP3^+^ Treg cells in colorectal cancer [38]. As for endometrial cancer, the Th17/Treg ratio in peripheral blood was reported to be significantly increased in endometrial cancer patients compared with healthy controls [39], suggesting similar mechanisms for the tumor-suppressing effect of Treg cells in this sort of malignancy as well. Further investigation is warranted to identify the underlying rationale for our findings on the prognostic significance of CD25^+^FOXP3^+^CD45RA^−^ Treg cells in endometrial carcinoma.

We previously reported that CD4^+^ TICs was found to be a significant and independent prognostic factor for favorable OS and to correlate with longer TFI [20], implicating that CD4^+^ helper T cells may affect patient prognosis through involvement in tumor chemosensitivity. Together with the present findings, among CD4^+^ helper T cell components, activated effector CD25^+^FOXP3^+^CD45RA^−^ T cells possibly play crucial roles in tumor chemosensitivity. High expressions of FOXP3 and CD25 are reported to be associated with chemoresistance in other sorts of malignancies [40–42], while not yet investigated in endometrial cancer. IL-2 treatment of colorectal cancer patients in a phase I/II peptide vaccination trial observed an expansion of peripheral CD4^+^CD25^high^FOXP3^+^ Treg cells [43]. Conversely, administration of CD25-blocking monoclonal antibody daclizumab led to significant and prolonged decrease in peripheral CD25^+^FOXP3^+^CD4 T cells in patients with metastatic breast cancer in the absence of autoimmunity [44]. Knockdown of macroH2A1, an epigenetic regulatory histone variant, in hepatocellular carcinoma cells promoted chemoresistance and CD4^+^CD25^+^FOXP3^+^ Treg cell activation [45]. In a prospective study on patients with metastatic breast cancer, treatment with CDK4/6 inhibitors (palbociclib, ribociclib or abemaciclib) in combination with hormonal therapy strongly reduced peripheral effector Treg subset (CD4^+^CD25^+^FOXP3^high^CD45RA^−^), the decrease of which was significantly greater in responder patients compared with non-responder patients [46]. Knockdown of HIF-1α blocked hypoxia-induced recruitment of CD4^+^CD25^+^ Treg cells in lung adenocarcinoma cell lines [17]. Immunization with FGF-2 peptides decreased CD4^+^CD25^+^FOXP3^+^ Treg cells and suppressed tumor vascularization and promoted apoptosis in grafted breast tumor mice model [18]. Although further verification is necessary for the exact prognostic role of CD25^+^FOXP3^+^CD45RA^−^ Treg cells in endometrial cancer, these lines of evidence together with our current findings suggest that the therapeutic manipulation of CD25^+^FOXP3^+^CD45RA^−^ effector Treg cells by antibodies, vaccines, or small molecules may promote tumor angiogenesis and chemosensitivity leading to improvement of patient prognosis in endometrial cancer.

Previous publications on the prognostic significance of Treg cells in endometrial cancer are limited, and the results are not consistent. Liu et al. analyzed the clinical and immunological characteristics of 575 endometrial carcinomas by utilizing the TCGA database, and showed that higher levels of Treg-cell infiltration were significantly associated with longer recurrence-free survival [47], keeping in line with our findings. Liu et al. screened the Treg-related gene signature by analyzing 522 endometrial cancers and 23 normal samples from the TCGA database, and reported significant correlations with better OS, higher TMB, and higher chemosensitivity [48], which are also consistent with the present findings. On the contrary, Chang et al. studied the phenotype expression of CD4^+^CD25^+^ Treg cells in the tumor-infiltrating lymphocytes of 57 endometrial carcinomas by flow cytometry, and reported a significant positive correlation with tumor grade, stage, and myometrial invasion [49]. De Jong et al. investigated the number of tumor-infiltrating CD8^+^ and FoxP3^+^ T-lymphocytes in 368 endometrial cancers by immunohistochemistry, and indicated that a high CD8^+^/FOXP3^+^ ratio was significantly associated with a better disease-free survival [50]. These different prognostic effects of Treg cells may be attributed to different analyzing methods and sorts of utilized samples. To the best of our knowledge, this is the first report on analyzing CD25^+^FOXP3^+^CD45RA^−^ effector Treg cells in endometrial cancer. The strengths of our study are the use of tumor tissues from patients treated by the consistent therapeutic strategy at a single institution and the application of cell-by-cell phenotyping technology by multiplexed immunofluorescence which can differentiate effector and naïve Treg cells in the stromal compartments specifically.

The current study contains a couple of limitations. First, this is a retrospective study which is possible to cause selection biases. Second, the sample size is relatively small. Third, the methods of evaluating immune cell components are different between CD25^+^, FOX3^+^, CTLA4^+^ or CD45RA^+^ cells and CD8^+^ cells. In spite of these, the results from bioinformatics analyses and multiple previous publications as described above support the validity and significance of our findings.

## Conclusions

We have demonstrated here that high CD25^+^, FOXP3^+^, and CD25^+^FOXP3^+^CD45RA^−^ stromal cell counts were associated with favorable OS, and that high CD25^+^ or high CD25^+^FOXP3^+^CD45RA^−^ stromal cell count was a significant and independent prognostic factor for favorable OS. The high ratio of CD25^+^ or CD25^+^FOXP3^+^CD45RA^−^ : total CD8^+^ TICs was each associated with longer TFI. Bioinformatics analyses showed that high mRNA expressions of CD25, FOXP3, CTLA4, and CD45RA correlated with better OS, and that high CD8^+^ T-cell infiltration and high CD25 / FOXP3 gene expression had the opposite prognostic effects. The current findings indicate that CD25^+^FOXP3^+^CD45RA^−^ effector Treg cells may be a useful prognostic biomarker and a potential target for manipulating tumor chemosensitivity to improve patient prognosis. We consider that further investigation will provide significant information for formulating novel immunotherapeutic strategies in advanced/recurrent endometrial carcinomas.

## Data Availability

The datasets used and/or analyzed during the present study are available from the corresponding author upon reasonable request.

## Abbreviations

FIGO: International Federation of Gynecology and Obstetrics
ICIs: Immune checkpoint inhibitors
MMR: Mismatch repair
MSI: Microsatellite instability
OS: Overall survival
TCGA: The Cancer Genome Atlas
TCR: T cell receptor
TFI: Treatment-free interval
TICs: Tumor-infiltrating immune cells
Treg: Regulatory T
TSA: Tyramide signal amplification
